# Arabidopsis Phosphatidic Acid Phosphohydrolases Are Essential for Growth under Nitrogen-Depleted Conditions

**DOI:** 10.3389/fpls.2017.01847

**Published:** 2017-10-31

**Authors:** Yushi Yoshitake, Ryoichi Sato, Yuka Madoka, Keiko Ikeda, Masato Murakawa, Ko Suruga, Daisuke Sugiura, Ko Noguchi, Hiroyuki Ohta, Mie Shimojima

**Affiliations:** ^1^School of Life Science and Technology, Tokyo Institute of Technology, Yokohama, Japan; ^2^Center for Biological Resources and Informatics, Tokyo Institute of Technology, Yokohama, Japan; ^3^Graduate School of Bioscience and Biotechnology, Tokyo Institute of Technology, Yokohama, Japan; ^4^Biomaterial Analysis Center, Technical Department, Tokyo Institute of Technology, Yokohama, Japan; ^5^Graduate School of Science, The University of Tokyo, Tokyo, Japan; ^6^School of Life Sciences, Tokyo University of Pharmacy and Life Sciences, Tokyo, Japan; ^7^Earth-Life Science Institute, Tokyo Institute of Technology, Tokyo, Japan

**Keywords:** phosphatidic acid phosphohydrolase, lipin, photosynthetic membrane, monogalactosyldiacylglycerol, nitrogen starvation, chloroplast, thylakoid membrane, *Arabidopsis thaliana*

## Abstract

The Arabidopsis homologs of mammalian lipin, PAH1 and PAH2, are cytosolic phosphatidic acid phosphohydrolases that are involved in phospholipid biosynthesis and are essential for growth under phosphate starvation. Here, *pah1 pah2* double-knockout mutants were found to be hypersensitive to nitrogen (N) starvation, whereas transgenic plants overexpressing PAH1 or PAH2 in the *pah1 pah2* mutant background showed a similar growth phenotype as compared with wild type (WT) under N starvation. The chlorophyll content of *pah1 pah2* was significantly lower than that of WT, whereas the chlorophyll content and photosynthetic activity of the transgenic plants were significantly higher than those of WT under N-depleted conditions. Membrane glycerolipid composition of the *pah1 pah2* mutants showed a significant decrease in the mole percent of chloroplast lipids to other phospholipids, whereas membrane lipid composition did not differ between transgenic plants and WT plants. Pulse-chase labeling experiments using plants grown under N-depleted conditions showed that, in *pah1 pah2* plants, the labeling percent of chloroplast lipids such as phosphatidylglycerol and monogalactosyldiacylglycerol in the total glycerolipids was significantly lower than in WT. Moreover, N starvation-induced degradation of chloroplast structure was enhanced in *pah1 pah2* mutants, and the membrane structure was recovered by complementation with PAH1. Thus, PAH is involved in maintaining chloroplast membrane structure and is required for growth under N-depleted conditions.

## Introduction

Nitrogen (N) is an essential macronutrient for plant growth and is used to produce many fundamental biological molecules such as nucleic acids, amino acids, proteins, and metabolites (Crawford and Forde, 2002; Peng et al., 2007). N starvation leads to severe growth retardation with concomitant decreases in the chlorophyll content and the efficiency of photosynthetic activities (Vidal and Gutiérrez, 2008; Maathuis, 2009; Boussadia et al., 2010). Thus, to enhance N uptake under N-depleted conditions, plants alter their lateral root architecture (Little et al., 2005; Orsel et al., 2006; Remans et al., 2006) and increase the expression of genes responsible for nitrate transport (Guo et al., 2001). Plants also enhance N remobilization from older to younger leaves and from reproductive organs (Ono et al., 1996; Hanaoka et al., 2002;Remans et al., 2006; Peng et al., 2007). Plant lipid composition also changes in response to N availability. Photosynthetic membranes, namely thylakoid membranes of chloroplasts, are predominantly composed of galactolipids such as monogalactosyldiacylglycerol (MGDG) and digalactosyldiacylglycerol (DGDG), which constitute ∼80% of the total thylakoid membrane lipids (Block et al., 1983). However, the galactolipid composition is greatly affected by N starvation. In soybean leaves, the content of MGDG and DGDG is decreased under N starvation, whereas phospholipid content remains unchanged during N starvation (Narasimhan et al., 2013). In Arabidopsis, the relative amount of MGDG in membrane lipids is decreased under N starvation (Gaude et al., 2007). The synthesis of fatty acid phytyl esters is enhanced in chloroplasts to avoid the accumulation of toxic intermediates, such as tetrapyrroles, free phytols, and free fatty acids, as a result of chlorophyll and galactolipid degradation during N starvation (Gaude et al., 2007). N starvation also increases triacylglycerol accumulation in vegetative tissues (Martin et al., 2002; Gaude et al., 2007; Yang et al., 2011). In Arabidopsis and *Brassica napus*, the increased expression of phospholipase D𝜀 (PLD𝜀), which hydrolyzes membrane phospholipids to produce the cellular signaling molecule phosphatidic acid, has the potential to improve plant growth under N-depleted conditions (Hong et al., 2009; Lu et al., 2016). Thus, genetic modifications to lipid synthesis pathways could potentially improve plant tolerance to N starvation. However, little is known about the detailed mechanism of N-starvation tolerance mediated by lipids.

The soluble phosphatidic acid phosphohydrolase, lipin, is involved in triacylglycerol biosynthesis and was originally identified in yeast and humans. Arabidopsis has two lipin homologs, PAH1 and PAH2 (Nakamura et al., 2009). Although Arabidopsis single-knockout mutants of PAH1 or PAH2 do not show any notable phenotype as compared with wild type (WT), the double-knockout mutant *pah1 pah2* has a higher level of phospholipids and a severe phenotype when grown under inorganic phosphate (Pi)-depleted conditions as compared with WT (Nakamura et al., 2009). Plants often suffer from Pi starvation because of the scarcity of inorganic Pi in soil, and thus they have multiple mechanisms to overcome Pi deficiency (Raghothama, 1999). One of these mechanisms is membrane lipid remodeling in which the phospholipid phosphatidylcholine in extraplastidial membranes is replaced with the non-phosphorus-containing galactolipid DGDG (Härtel and Benning, 2000; Dörmann and Benning, 2002). One important membrane lipid remodeling pathway produces diacylglycerol (DAG), which is a substrate for MGDG synthase. During Pi starvation, galactolipid synthesis is upregulated via the activation of type B MGDG synthase (MGD2 and MGD3 in Arabidopsis), which is localized in the outer envelope membrane of chloroplasts (Awai et al., 2001; Kobayashi et al., 2009). The DAG generated via phospholipid degradation is supplied to the type B MGDG synthase, and the resultant MGDG is subsequently used for DGDG synthesis (Shimojima and Ohta, 2011; Nakamura, 2013). Because *pah1 pah2* plants show severe growth retardation under Pi-depleted conditions, PAH1 and PAH2 are considered to be key enzymes in Pi starvation-induced lipid remodeling, which plays a role in releasing Pi via phospholipid degradation (required for other essential biological processes) and in supplying DAG for galactolipid synthesis as a substrate of MGDG synthase (Nakamura et al., 2009).

Here, we found that Arabidopsis *pah1 pah2* mutants showed a severe growth phenotype, whereas transgenic plants overexpressing PAH1 or PAH2 in the *pah1 pah2* background showed slightly higher photosynthetic activity under N starvation as compared with WT. Our results showed that PAH1 and PAH2 are involved not only in phospholipid homeostasis in the endoplasmic reticulum (ER) but also in the maintenance of the thylakoid membrane in chloroplasts and its photosynthetic activity, especially under N starvation.

## Materials and Methods

### Plant Material and Growth Conditions

The isolation of the *Arabidopsis thaliana pah1 pah2* mutant and the generation of transgenic plants overexpressing PAH1 or PAH2 and harboring GFP in a *pah1 pah2* background were previously described (Nakamura et al., 2009). Surface-sterilized seeds of WT *A. thaliana* (Columbia-0), the *pah1 pah2* mutant, and the transgenic mutant lines were incubated at 4°C in darkness for 3 days prior to plating on Murashige and Skoog medium (Murashige and Skoog, 1962) containing 0.8% (w/v) agar supplemented with 1% (w/v) sucrose. Plants were then incubated at 22°C under continuous white light (40–50 μmol m^-2^ s^-1^) for all growth conditions. Arabidopsis seeds were grown on solidified Murashige and Skoog agar supplemented with 1% (w/v) sucrose for 10 days and then were grown for another 7 days on a solidified N-sufficient (4.5 mM N) or N-depleted (0 mM N) medium (Estelle and Somerville, 1987; Gaude et al., 2007) supplemented with 1% (w/v) sucrose, with KNO_3_ and Ca(NO_3_)_2_⋅4H_2_O replaced with KCl_2_ and CaCl_2_, respectively.

### Quantitative Reverse Transcription-PCR

Total RNA was isolated from three independent plant samples using the SV Total RNA Isolation System (Promega). Reverse transcription was performed using the PrimeScript RT reagent kit (TaKaRa Bio), and cDNA amplification was carried out using SYBR PreMix Ex Taq (TaKaRa Bio). Signal detection and quantification were performed in duplicate using the Thermal Cycler Dice Real Time System (TaKaRa Bio). Quantitative PCR determination of *MGD1, MGD2, MGD3, DGD1, DGD2, PES1*, and *PES2* transcripts was normalized using the Arabidopsis *UBQ10* transcript level (Sun and Callis, 1997). Expression levels were obtained from at least three replicates. The gene-specific primers used were as follows: PAH1 Fw (5′ GGATAACGAGGACAGGAAGACTG 3′); PAH1 Rv (5′ AGCAGCTGCGCTAAGTCCCATAC 3′); PAH2 Fw (5′ CTCAAGCCTCAGTCACAAGACAA 3′); PAH2 Rv (5′ AAGGAAAGAGACCATCAGGAGAGA 3′); PES1 Fw (5′ CCTGTCACCGCAACCAATC 3′); PES1 Rv (5′ ATTGTTGCACCAAACCGTGCT 3′); PES2 Fw(5′ CTCTTCTCCTATACTTACCTGG 3′); PES2 Rv (5′ CCTCAATAAGCTTCACCAAGT 3′); MGD1 Fw (5′ AGGTTTCACTGCGATAAAGTGGTT 3′); MGD1 Rv (5′ AACGGCAATCCCTCCTCAC 3′); MGD2 Fw (5′ GATTCGATCACTTCCTATCATCCTC 3′); MGD2 Rv (5′ TGTGCTAAACCATTCCCCAAC 3′); MGD3 Fw (5′ TCGTGGCGGATTGGTTTAG 3′); MGD3 Rv (5′ CGTTGTTGTTGTTGGGATAGATG 3′); DGD1 Fw (5′ CTGAAGAGAGATCCCGTGGTG 3′); DGD1 Rv (5′ TCCCAAGTTCGCTTTTGTGTT 3′); DGD2 Fw(5′ TGCAGAACCTATGACGATGGA 3′); DGD2 Rv (5′ GCTCTGTAAGTTGCGATGGTTG 3′); UBQ10 Fw (5′ GGCCTTGTATAATCCCTGATGAATAAG 3′); UBQ10 Rv (5′ AAAGAGATAACAGGAACGGAAACATAGT 3′).

### Measurement of Chlorophyll Content

The 17-day-old seedlings were incubated in 1 mL dimethylformamide (Wako) at 4°C for 20 h in the dark, and then absorbance was measured at 646.8 and 663.8 nm. Chlorophyll content was calculated as follows: Chl*a* = (13.43A_663.8_ – 3.47A_646.8_)/sample fresh weight, and Chl*b* = (22.9A_646.8_ – 4.88A_663.8_)/sample fresh weight (Moran and Porath, 1980).

### Analysis of Chlorophyll Fluorescence

The maximum quantum efficiency (*F*_v_/*F*_m_) of photosystem II was measured using a Dual-PAM system (Walz). The minimum chlorophyll fluorescence (*F*_o_) was determined by measuring light (intensity set at 20). After the *F*_o_ determination, a saturating pulse of red light (intensity set at 15) was applied to determine the maximum chlorophyll fluorescence (*F*_m_). *F*_v_/*F*_m_ was calculated as (*F*_m_ – *F*_o_)/*F*_m_. Before the *F*_v_/*F*_m_ measurements, samples were incubated for 10 min in the dark. Samples were in plastic plates when measured.

### Determination of N Concentration

To determine N concentration, ∼0.1 g fresh weight of shoots from 10- or 17-day-old seedlings was washed and oven dried at 80°C for a minimum of 3 days and then ground into powder. The N concentration was determined using a CN analyzer (Vario ELIII; Elementar Analysensysteme GmbH) according to Hachiya and Noguchi (2008).

### Electron Microscopy

Leaf segments were fixed with 2% (w/v) paraformaldehyde and 2.5% (w/v) glutaraldehyde in 0.067 M phosphate buffer (pH 7.4) for 2 h at room temperature and then for 16 h at 4°C. Samples were then washed six times in the phosphate buffer for 10 min each at room temperature. They were post-fixed with 2% (w/v) osmium tetroxide in 0.067 M phosphate buffer (pH 7.4) for 2 h at room temperature. The fixed samples were dehydrated in a graded ethanol series and embedded in an epoxy resin mixture (Quetol 651 mixture; Nisshin EM). Ultrathin 70-nm sections were cut with a diamond knife on a Leica EM-UC7 ultramicrotome and were transferred to copper grids. The sections were stained with EM stainer (Nisshin EM) for 1 h, followed by Reynolds’s lead citrate for 9 min at room temperature. The specimens were observed with a JOEL JEM-1400 Plus transmission electron microscope at an accelerating voltage of 80 kV.

### Lipid Analysis

Total lipids were extracted from tissues as described (Bligh and Dyer, 1959). Polar membrane lipids were separated by two-dimensional thin-layer chromatography (Kobayashi et al., 2007). Neutral glycerolipids were separated by one-dimensional thin-layer chromatography using the solvent system of hexane/diethyl ether/acetic acid (160:40:4, v/v/v). Lipids on silica gel plates were visualized with 0.01% (w/v) primuline in 80% (v/v) acetone under UV light. Lipids isolated from silica gel plates were methylated, and fatty acid methyl esters were quantified by gas chromatography using pentadecanoic acid as an internal standard (Kobayashi et al., 2006).

### Pulse-Chase Labeling Experiments

*In vivo* labeling experiments with [^14^C]acetate were conducted according to Nakamura et al. (2009), except that [^14^C]acetate was diluted to 0.05 mCi mL^-1^ with N-depleted medium (Estelle and Somerville, 1987; Gaude et al., 2007). [^14^C]acetate was spread with a micropipette in 3-μL droplets over the surface of the third and fourth leaves cut off from 15-day-old seedlings, and the leaves were placed on 3MM paper immersed in N-depleted medium and incubated for 2 h at 22°C. After the incubation, the leaves were washed twice in non-radioactive N-depleted medium to remove exogenous radioactivity and were placed on 3MM paper immersed in N-depleted medium. After the incubation, the samples were harvested and were frozen in liquid nitrogen. Lipids were extracted and separated by two-dimensional TLC as described above. Radioactive spots were analyzed by autoradiography (FLA7000, Fuji Film) and Imaging Plate (Fuji Film).

## Results

### The *pah1 pah2* Mutants Are Hypersensitive to N Starvation

**FIGURE 1:**
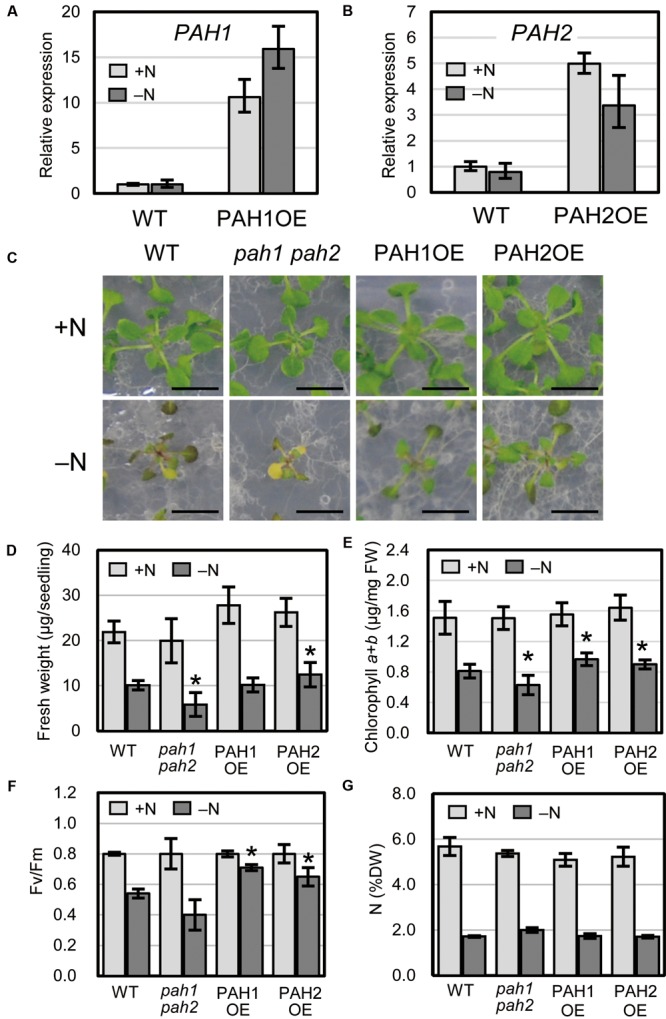
Expression of *PAH1* and *PAH2* in the transgenic lines and the effect of N starvation on Arabidopsis growth. **(A)** Expression of *PAH1* in PAH1OE, **(B)** expression of *PAH2* in PAH2OE, **(C)** growth phenotypes, **(D)** shoot fresh weight, **(E)** chlorophyll content, **(F)** *F*_v_/*F*_m_, and **(G)** total N concentration in shoots. WT, *pah1 pah2*, PAH1OE, and PAH2OE were grown under N-sufficient (+N, light gray) and N-depleted (-N, dark gray) conditions. Scale bars: 1 cm. Data represent the mean ± SD from three independent experiments; ^∗^*P* < 0.05 for a *t*-test versus WT under each condition. FW, fresh weight; DW, dry weight.

We previously produced Arabidopsis transgenic plants overexpressing PAH1 or PAH2 in the *pah1 pah2* background (Nakamura et al., 2009). Expression levels of *PAH1* and *PAH2* were analyzed for several lines for each transgenic combination, and PAH1OE 20-1 and PAH2OE 22-1 were selected and used as PAH1OE and PAH2OE, respectively, for further analyses (**Supplementary Figure S1**). Expression of *PAH1* in PAH1OE and *PAH2* in PAH2OE was 11-fold and 5-fold higher than that in WT, respectively, under normal growth conditions (+N), and these levels were 16-fold and 3-fold higher than that in WT, respectively, under N starvation (–N) (**Figures 1A,B**). When we grew plants under N-depleted conditions, seedlings of the Arabidopsis double-knockout mutant *pah1 pah2* were significantly smaller than WT seedlings, whereas PAH1OE and PAH2OE seedlings were comparable to or larger than WT seedlings (**Figures 1C,D**). Under N-sufficient conditions, the chlorophyll content in WT, *pah1 pah2*, PAH1OE, and PAH2OE did not differ significantly (**Figure 1E**). Under N-depleted conditions, however, the chlorophyll content in *pah1 pah2* was significantly lower than that in WT, whereas the content in PAH1OE and PAH2OE was similar to or greater than that in WT (**Figure 1E**). As for photosynthetic activity parameters under N-depleted conditions, both PAH1OE and PAH2OE showed significantly higher maximum quantum yield (*F*_v_/*F*_m_) as compared with those of WT and *pah1 pah2* (**Figure 1F**). The differences in sensitivity to N starvation among WT, *pah1 pah2*, PAH1OE, and PAH2OE could be due to the enhanced efficiency of N uptake during the first 10 days of growth under N-sufficient conditions. Therefore, we assessed total N concentration in seedlings of WT, *pah1 pah2*, PAH1OE, and PAH2OE; the N concentrations across these plants were comparable under both N-sufficient and N-depleted conditions (**Figure 1G**), suggesting that the growth defect in *pah1 pah2* during N starvation is not due to the lower N content in the seedlings as compared with the other plants.

### Chloroplast Membrane Structure Was Severely Disrupted in *pah1 pah2* under N-Depleted Conditions

**FIGURE 2:**
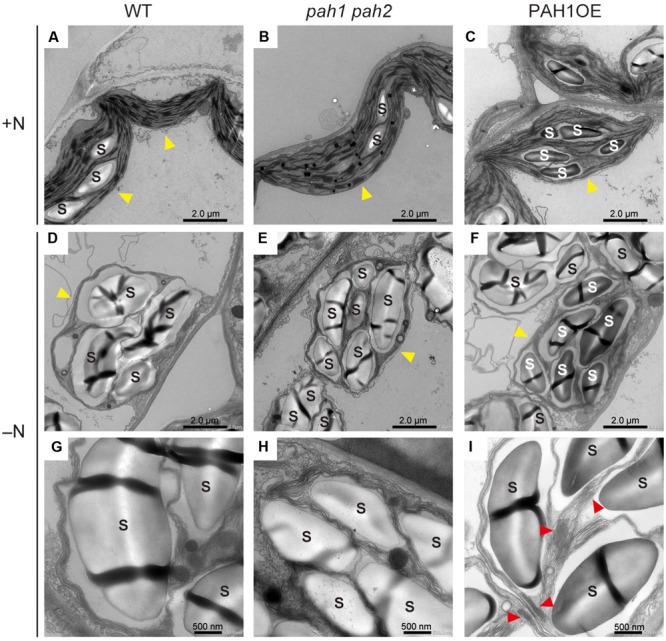
Electron microscopy of chloroplast membrane structures in leaves. **(A)** WT, **(B)** *pah1 pah2*, and **(C)** PAH1OE under N-sufficient conditions (+N). **(D)** and **(G)** WT, **(E)** and **(H)** *pah1 pah2*, and **(F)** and **(I)** PAH1OE under N-depleted conditions (-N). Red arrowheads in **(I)** indicate grana-lamellae structures in thylakoid membranes. Yellow arrowheads indicate chloroplasts. S, starch granules.

As a significant decrease in chlorophyll content was observed only in *pah1 pah2* grown under N starvation, we visualized the intracellular structure of plant leaves with electron microscopy and compared the structures among WT, *pah1 pah2*, and PAH1OE under N-sufficient and N-depleted conditions (**Figure 2** and **Supplementary Figure S2**). Under N-sufficient conditions, chloroplast membrane structures were similar among WT, *pah1 pah2* and PAH1OE (**Figures 2A–C**). Under N-depleted conditions, starch accumulation in chloroplasts was observed across all of the plants (**Figures 2D–F** and **Supplementary Figure S2**). The belt shaped-structures observed in starch granules have no biological phenotype but the technical reason of cutting the sample in the resin for microscopic analysis. As for the thylakoid membrane structures in the enlarged images, however, we could often observe grana-lamellae stacking structures in the thylakoid membrane of WT and PAH1OE but not in *pah1 pah2* under N-depleted conditions (**Figures 2G–I** and **Supplementary Figure S2**). Thus, it was clearly shown that the absence of PAH enhances breakdown of the chloroplast membrane structure, especially under N starvation and thus decreases chlorophyll content under N-depleted conditions as compared with WT (**Figure 1E**). We also observed the chloroplast membrane structure of PAH1OE. The membrane structure of PAH1OE was either comparable with WT or showed a slightly increased number of grana-lamellae stacking structures under N-depleted conditions (**Figures 2C,F,I** and **Supplementary Figure S2**). Although we could not assess these data statistically because the number of grana-lamellae stacking structures in *pah1 pah2* was markedly lower than that in WT and PAH1OE, the stacking repeat distance in thylakoids of *pah1 pah2* was wider than those in WT and PAH1OE (**Supplementary Figure S3**). Thus, our results showed that the absence of PAH affects the structure of photosynthetic membranes in chloroplasts, especially under N starvation.

### The Mole Percent of Chloroplast Lipids Was Markedly Decreased in the *pah1 pah2* Mutant under N Starvation

**FIGURE 3:**
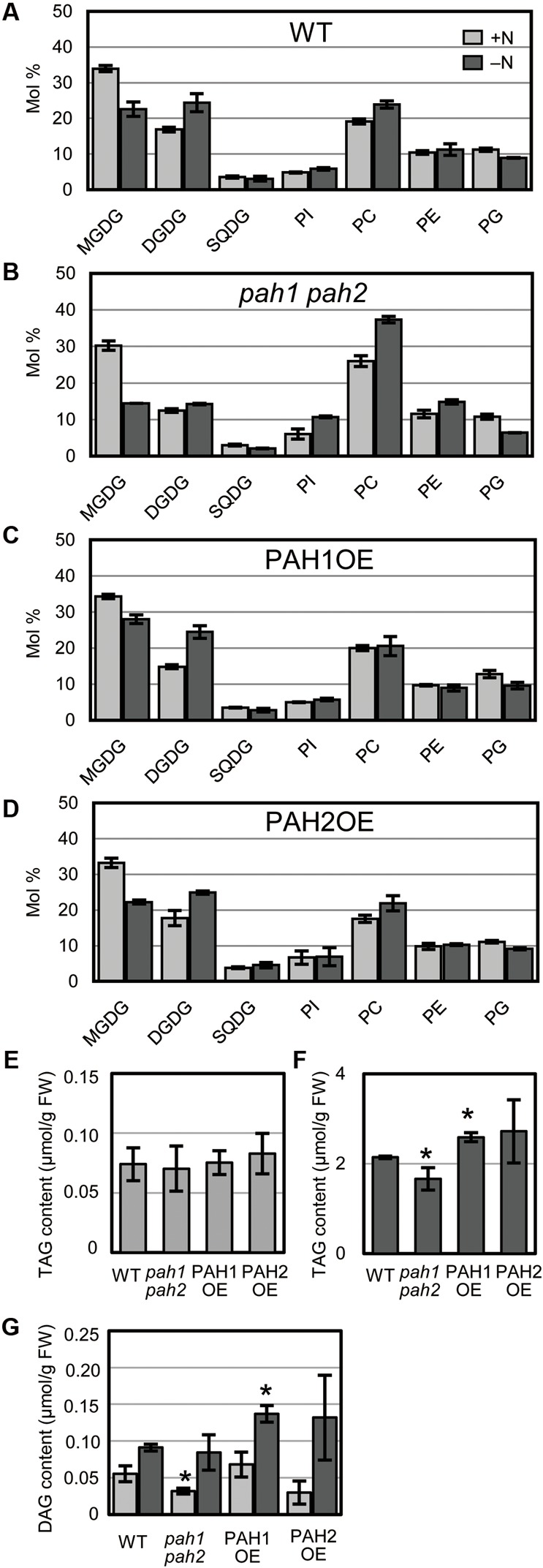
Glycerolipid composition. Membrane glycerolipid composition of **(A)** WT, **(B)** *pah1 pah2*, **(C)** PAH1OE, and **(D)** PAH2OE. The TAG contents under N-sufficient conditions **(E)** and under N-depleted conditions **(F)**. **(G)** The DAG content. Results for N-sufficient (+N, light gray) and N-depleted (-N, dark gray) conditions are shown. SQDG, sulfoquinovosyldiacylglycerol; PI, phosphatidylinositol; PE, phosphatidylethanolamine; PG, phosphatidylglycerol; TAG, triacylglycerol; DAG, diacylglycerol. Values represent the mean ± SD of measurements made on samples from three different plants for each genotype. ^∗^*P* < 0.05 for a *t*-test versus WT under each condition.

Nitrogen starvation leads to a decrease in MGDG and a concomitant increase in DGDG in WT Arabidopsis (Gaude et al., 2007). In *pah1 pah2*, the mole percent of MGDG and DGDG in the total glycerolipids are smaller than in WT, even under N-sufficient conditions (Nakamura et al., 2009), which was also observed in our results (**Figure 3**). However, in *pah1 pah2*, the mole percent of MGDG decreased notably whereas that of DGDG only slightly increased during N starvation as compared with those in WT, PAH1OE, and PAH2OE (**Figure 3**). In PAH1OE and PAH2OE, the membrane lipid compositions were similar to one another, and changes in their mole percent during N starvation did not differ substantially from WT, except that the mole percent of MGDG in PAH1OE was slightly greater than that in WT under N-depleted conditions (**Figures 3A,C,D**). Given that PAH1 and PAH2 similarly complemented the *pah1 pah2* phenotype with respect to membrane glycerolipid composition (**Figures 3A,C,D**), PAH1 and PAH2 may have a redundant role in maintaining membrane lipid homeostasis of the ER and chloroplasts under both N-sufficient and N-depleted conditions. Moreover, these results clearly show that PAH1 and PAH2 play an important role in maintaining the mole percent of chloroplast membrane lipids to the extraplastidial membrane lipids, namely phospholipids, especially under N-depleted conditions. Given that the fatty acid compositions of MGDG and DGDG were similar among WT, *pah1 pah2*, PAH1OE and PAH2OE under both N conditions (**Supplementary Figure S4**), it was suggested that the prokaryotic pathway and the eukaryotic pathway equally contributed to the galactolipid synthesis. We also analyzed the TAG and DAG contents (**Figures 3E–G**). Under N-sufficient conditions, the amounts of TAG were comparable among plants (**Figure 3E**). However, under N-depleted conditions, the amount of TAG in *pah1 pah2* was lower and that in PAH1OE was slightly higher than that in WT (**Figure 3F**), suggesting that PAH1 is involved in the TAG accumulation in leaves under N starvation. PAH2OE showed a similar result to PAH1OE, but it was not significant. Given that the fatty acid compositions of TAG were similar among WT, *pah1 pah2*, PAH1OE and PAH2OE under both N conditions (**Supplementary Figures S5A–D**), it was suggested that PAH1 (and PAH2) might be only slightly involved in TAG synthesis under N starvation. The DAG contents also showed similar profile with the TAG contents (**Figure 3G**). Compared with WT, the lower amount of DAG in *pah1 pah2* under N-sufficient conditions and the higher amount of DAG in PAH1OE under N-depleted conditions were observed (**Figure 3G**). However, the fatty acid compositions of DAG were varied in *pah1 pah2*, PAH1OE and PAH2OE (**Supplementary Figures S5E–H**), suggesting that the lack or overexpression of PAH might destabilize the whole flow of DAG synthesis.

### Expression of the Genes for Fatty Acid Phytyl Ester Synthase in *pah1 pah2* Was Higher than in Other Plants under N Starvation

**FIGURE 4:**
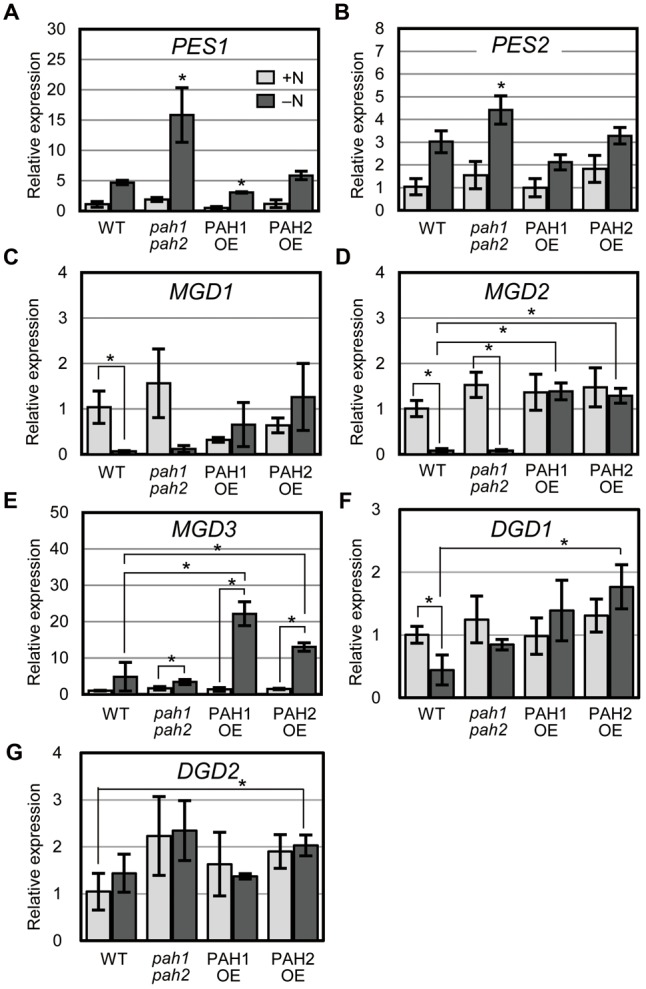
Expression of genes encoding fatty acid phytyl ester synthase, MGDG synthase and DGDG synthase. Expression of **(A)** *PES1*, **(B)** *PES2*, **(C)** *MGD1*, **(D)** *MGD2*, **(E)** *MGD3*, **(F)** *DGD1* and **(G)** *DGD2* in plants of the indicated genotype relative to their corresponding levels in WT under N-sufficient conditions as assessed with quantitative reverse transcription-PCR. N-sufficient (+N, light gray) and N-depleted (-N, dark gray) conditions are shown. Values represent the mean ± SD of measurements made on samples from three different plants for each genotype. **(A,B)** ^∗^*P* < 0.05 for a *t*-test versus WT under each condition. **(C–G)** ^∗^*P* < 0.05 for a *t*-test between plants indicated by the brackets.

In plants grown under N-depleted conditions, the synthesis of fatty acid phytyl esters catalyzed by PES1 and PES2 is accelerated in chloroplasts to avoid the accumulation of free fatty acids and phytols derived from the degradation of thylakoid membrane lipids and chlorophyll, respectively (Lippold et al., 2012). Thus, we analyzed the expression of *PES1* and *PES2* under N-sufficient and N-depleted conditions (**Figures 4A,B**). In WT, the expression of *PES1* and *PES2* increased markedly under N-depleted conditions (Lippold et al., 2012) (**Figures 4A,B**). In *pah1 pah2*, the expression of *PES1* and *PES2* was significantly higher than that in WT under N-depleted conditions (**Figures 4A,B**), suggesting that the breakdown of MGDG and chlorophyll was enhanced in *pah1 pah2*, especially during N starvation, which is consistent with the results in **Figure 1**. In contrast, in PAH1OE and PAH2OE, the expression of *PES1* and *PES2* was comparable with that in WT under both N-sufficient and N-depleted conditions (**Figures 4A,B**).

### Expression of MGDG Synthase Genes Was Comparable between WT and *pah1 pah2* but Was Upregulated in PAH1OE and PAH2OE Especially under N-Depleted Conditions

To clarify the effect of the absence or the overexpression of PAH on galactolipid synthesis, we analyzed the expression of MGDG synthesis genes (*MGD1, MGD2*, and *MGD3*, Awai et al., 2001) and DGDG synthesis genes (*DGD1* and *DGD2*, Dörmann et al., 1999; Kelly and Dörmann, 2002) and compared their expression levels among WT, *pah1 pah2*, PAH1OE, and PAH2OE under both N conditions (**Figures 4C–G**). In both WT and *pah1 pah2*, the expression of *MGD1* and *MGD2* was significantly decreased during N starvation, whereas it remained unchanged in PAH1OE and PAH2OE during N starvation (**Figures 4C,D**). In WT and *pah1 pah2, MGD3* expression remained unchanged or slightly increased during N starvation, whereas its expression in PAH1OE and PAH2OE under N starvation was significantly higher than that under normal conditions (**Figure 4E**). Thus, although the three genes for MGDG synthesis showed mostly higher expression in PAH1OE and PAH2OE than in WT and *pah1 pah2* during N starvation, these expression levels were not remarkably different between WT and *pah1 pah2* under any N conditions. On the other hand, although the expression of *DGD1* in PAH1OE and PAH2OE under N-depleted conditions seemed higher than that in WT and *pah1 pah2* (**Figure 4F**), most of the expression was similar among plants under any conditions (**Figures 4F,G**). These results indicated that the absence of PAH has no effect on the expression of MGDG synthases under any N conditions, but the overexpression of PAH increases their expression levels, especially during N starvation.

### Pulse-Chase Labeling of Membrane Lipids during N Starvation Indicated a Significant Decrease in the Labeling Percent of Chloroplast Lipids in *pah1 pah2*

**FIGURE 5:**
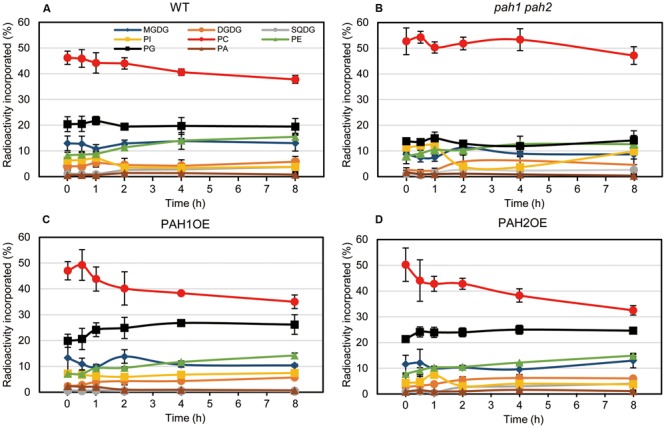
*In vivo* pulse-chase labeling of membrane lipids during N starvation. [^14^C]acetate labeling of fatty acids associated with individual lipids in **(A)** WT, **(B)** *pah1 pah2*, **(C)** PAH1OE, and **(D)** PAH2OE under N-depleted conditions was analyzed. Experiments were repeated three times, each with similar results. Values represent the mean ± SD of measurements made on samples from three different plants for each genotype.

In the case of [^14^C]acetate labeling of Arabidopsis WT seedlings under normal growth conditions, the ^14^C label is rapidly incorporated into PC and MGDG (Browse et al., 1986; Xu et al., 2003; Nakamura et al., 2009). Under N-depleted conditions, as well as under N-sufficient conditions, PC was the major ^14^C-labeled lipid. However, the second most prevalent labeled lipid was PG in WT (**Figure 5A**). In *pah1 pah2*, the labeling percent of PC in the total labeled glycerolipids was markedly higher than that of WT (**Figure 5B**), which was plausibly caused by the enhanced PC synthesis in *pah1 pah2*, even under N starvation, or by the repression of PC breakdown and lipid trafficking from the ER to chloroplasts. Accordingly, in *pah1 pah2*, the relative amounts for labeled chloroplast lipids such as MGDG and PG in the total labeled glycerolipids were lower than in WT (**Figure 5B**). However, it is noteworthy that the percent of labeled phosphatidylinositol (PI) in *pah1 pah2* was about 2-fold higher than that in WT at the beginning and end of the time course (**Figures 5A,B**), which is consistent with the membrane glycerolipid compositions under N starvation in *pah1 pah2* (**Figure 3B**). In PAH1OE and PAH2OE, the profile of the labeled lipids, except PC and PG, were comparable with that in WT throughout the time course (**Figures 5A,C,D**). In PAH1OE and PAH2OE, the percent of the labeled PC was decreased by ∼8% during the initial 2 h, whereas the decrease was <5% in WT (**Figures 5A,C,D**). Moreover, the percent of the labeled PG increased by ∼5% over the time course, whereas that in WT and *pah1 pah2* remained unchanged (**Figure 5**). However, the percent of PC and PG in the membrane glycerolipids were not significantly different among WT, PAH1OE, and PAH2OE (**Figures 3A,C,D**). Thus, the exchange of the labeled fatty acids between PC and PG might occur in PAH1OE and PAH2OE under N starvation, but the amount is too small to affect the membrane glycerolipid composition.

## Discussion

We previously reported that PAH is involved in Pi-starvation tolerance, because the growth of *pah1 pah2* is severely impaired under Pi starvation (Nakamura et al., 2009). In the *pah1 pah2* mutant, the relative amount of galactolipids to phospholipids is lower than in WT under both Pi-sufficient and Pi-depleted conditions owing to an enhanced PC synthesis in the ER and a decreased supply rate of DAG from the ER to chloroplasts (Nakamura et al., 2009; Eastmond et al., 2010; Craddock et al., 2015, 2016). Thus, the negative effect on growth during Pi starvation can be explained simply as a decrease in the amount of Pi released from phospholipid degradation caused by the absence of PAH. However, here we found that PAH was also essential for growth under N starvation. PAH is involved in PC and phosphatidylethanolamine (PE) degradation because the substrate for PAH is PA produced from PC or PE by PLD. Thus, we first thought that N released from phospholipids could be essential for maintaining the N content in the cell and its growth under N starvation. However, compared with the amount of N in chlorophyll and proteins, that in phospholipids such as PC, PE, and phosphatidylserine is too low to suggest that phospholipids function in N storage (Carrari et al., 2005; Gaude et al., 2007). Indeed, the N content in the seedlings was comparable between WT and *pah1 pah2* under either N condition. Thus, the severe phenotype of *pah1 pah2* under N starvation was not caused by a decrease in the N content, and the absence of PAH had no effect on the N content in the seedlings.

PA accumulates in *pah1 pah2* seedlings (Nakamura et al., 2009). As PA is a signaling molecule for some environmental stresses (Katagiri et al., 2001; Munnik, 2001; Sang et al., 2001; Jouhet et al., 2012), it is also possible that the accumulation of PA in *pah1 pah2* during N starvation affects the signaling cascade related to N starvation. However, overexpression of PLD𝜀 enhances N signaling and growth in Arabidopsis and *Brassica napus*, and PA produced by PLD𝜀 functions as a lipid messenger response to N starvation (Hong et al., 2009; Lu et al., 2016). In Arabidopsis, there are 12 PLDs that differ with respect to their localization and functions (Testerink and Munnik, 2011). It is not known which PLD is involved in the PA supply for PAH, but it is most likely that differences in the localization and the timing of PA accumulation result in the different effects on plant growth. Thus, to clarify the correlation between PA content as a signaling molecule and tolerance to N starvation in *pah1 pah2*, we will need to undertake additional experiments using transgenic plants—resulting from crosses between *pah1 pah2* mutants, PAH-overexpressing plants, PLD mutants or overexpressing plants—that have been modified with respect to PA content and to the subcellular localization of PA. Recently, the involvement of phosphatidylinositol–phospholipase C (PI-phospholipase C) in the N signaling pathway was shown in Arabidopsis (Riveras et al., 2015). PI-phospholipase C affects the amount of calcium ions in the cytosol, and the resulting increase in calcium ions activates the expression of N-responsive genes. PIphospholipase C produces DAG and inositol 1,4,5-triphosphate from PI. In *pah1 pah2* during N starvation, not only the mole percent of PC but also that of PI was remarkably higher than WT in the membrane glycerolipids, suggesting that the 1,4,5-triphosphate synthesis from PI might be affected as well as the synthesis of other phospholipids under N starvation.

In Arabidopsis WT seedlings, a marked decrease in the MGDG mole percent with an increase in the DGDG mole percent in the total glycerolipids occurs during N starvation (Gaude et al., 2007), which was also observed in our results. However, in *pah1 pah2*, the decrease in the MGDG mole percent was even greater during N starvation as compared with WT. As these results were based on the relative amount of the lipids, it seemed that the results might simply be explained by the enhancement of PC synthesis in *pah1 pah2* (Eastmond et al., 2010). However, the microscopic analysis clearly showed a difference in membrane structures between WT and *pah1 pah2*. The enhanced degradation of the thylakoid membranes was observed only in *pah1 pah2* grown under N starvation, indicating that PAH is involved in the maintenance of thylakoid membranes during N starvation. Indeed, in *pah1 pah2*, fatty acid phytyl ester synthesis was transcriptionally upregulated, especially under N starvation, suggesting that the degradation of MGDG is enhanced in *pah1 pah2* during N starvation. PAH was previously described as not being involved in lipid trafficking between the ER and chloroplast and as being involved only in phospholipid synthesis and its homeostasis in the ER under normal growth conditions (Eastmond et al., 2010). Under Pi-depleted conditions, however, we observed the involvement of PAH in lipid trafficking between the ER and chloroplast based on the difference in the fatty acid composition of MGDG, although its contribution to lipid trafficking seemed smaller than expected considering the severe phenotype of *pah1 pah2* under Pi starvation (Nakamura et al., 2009). Under N starvation, most of the lipid synthetic pathways except PC synthesis was markedly downregulated in *pah1 pah2* based on the results from our pulse-chase labeling experiments. Thus, it is unclear that PAH is involved in lipid trafficking between the ER and chloroplast under N starvation as well as under Pi starvation.

We previously produced *pah1 pah2* complementation lines (Nakamura et al., 2009), which overexpress PAH1 or PAH2 and were designated as PAH1OE or PAH2OE, respectively, in this study. PAH1OE and PAH2OE complemented the *pah1 pah2* phenotype, especially with respect to membrane lipid composition and growth under N-sufficient and N-depleted conditions. However, one of the photosynthetic parameters, *F*_v_/*F*_m_, and the chlorophyll content were significantly greater in both PAH1OE and PAH2OE as compared with WT, especially under N starvation. These results clearly showed that PAH affects the photosynthetic activity and the chlorophyll content during N starvation. Although the membrane glycerolipid composition of these two overexpressing lines was comparable with WT, enhancement of the DAG supply from the ER to chloroplasts might be a trigger for enhancement of membrane lipid turnover in chloroplasts, because DAG is a common substrate for the synthesis of the lipids MGDG, sulfoquinovosyldiacylglycerol, and PG. Indeed, in the pulse-chase experiments using [^14^C]acetate during N starvation, the most remarkable finding was that PG was the second-most predominant labeled lipid during N starvation, which has not been observed under normal growth conditions. Moreover, the labeling percents were increased only in PAH1OE and PAH2OE throughout the time course. Thus, it may be that the plants are dying under N starvation but manage to maintain PG synthesis because PG is the essential lipid for photosynthesis (Hagio et al., 2002; Kobayashi et al., 2015, 2016; Lin et al., 2016). In *pah1 pah2*, the labeling percent of PG and the other chloroplast lipids was significantly lower than WT, possibly because of the enhanced PC synthesis in *pah1 pah2* and the decrease in PC breakdown even under N starvation. The enhanced incorporation of [^14^C]acetate into PC might cause the reduced incorporation of [^14^C]acetate into the chloroplast membrane lipids. In contrast, in PAH1OE and PAH2OE, the labeling percent of PG increased with a concomitant decrease in that of PC during N starvation, which was not observed in WT. Although PAH might have a crucial role for regulating lipid homeostasis between the ER and chloroplasts, the molecular mechanism was not clarified in this study. The yet unknown molecular mechanism might explain why the expression of *MGD2* and *MGD3* in PAH1OE and PAH2OE was markedly higher than in WT, especially under N starvation.

## Author Contributions

YY, KN, HO, and MS directed the study. YY and MS designed the experiments. YY, RS, YM, KI, MM, KS, and DS performed the experiments and analyzed the data. YY and MS wrote the manuscript.

## Conflict of Interest Statement

The authors declare that the research was conducted in the absence of any commercial or financial relationships that could be construed as a potential conflict of interest.

## Supplementary Material

FIGURE S1 Expression levels of *PAH1* and *PAH2* in WT, *pah1 pah2*, and the transgenic plants PAH1OE and PAH2OE under N-sufficient conditions. Expression of *PAH1* and *PAH2* relative to their corresponding levels in WT under N-sufficient conditions as assessed by quantitative reverse transcription–PCR. Values represent the mean ± SD of measurements made on samples from three different plants for each genotype.

FIGURE S2 Electron microscopy of chloroplast membrane structures in leaves. **(A,D)** WT, **(B,E)** *pah1 pah2*, and **(C,F)** PAH1OE under N-depleted conditions. Red arrowheads indicate grana-lamellae stacking structures in thylakoid membranes. S, starch granules.

FIGURE S3 Ultrastructural analysis of the stacking repeat distance in thylakoids of WT, *pah1 pah2*, and PAH1OE under N-depleted conditions. **(A)** WT, **(B)** *pah1 pah2*, **(C)** PAH1OE. The repeat distance of grana thylakoid membranes was analyzed from transmission electron microscopy images according to the method of Wang et al. (2014). **(A)** and **(C)**, *n* = 100 grana thylakoid membrane regions from 10 different chloroplasts, with 10 grana thylakoid membrane regions from each chloroplast; **(B)**, *n* = 42 grana thylakoid membrane regions from 10 different chloroplasts, with four or five grana thylakoid membrane regions from each chloroplast.

FIGURE S4 Fatty acid compositions of MGDG and DGDG. MGDG in **(A)** WT, **(B)** *pah1 pah2*, **(C)** PAH1OE, and **(D)** PAH2OE. DGDG in **(E)** WT, **(F)** *pah1 pah2*, **(G)** PAH1OE, and **(H)** PAH2OE. Results for N-sufficient (+N, light gray) and N-depleted (-N, dark gray) conditions are shown. Values represent the mean ± SD of measurements made on samples from three different plants for each genotype.

FIGURE S5 Fatty acid compositions of TAG and DAG. TAG in **(A)** WT, **(B)** *pah1 pah2*, **(C)** PAH1OE, and **(D)** PAH2OE. DAG in **(E)** WT, **(F)** *pah1 pah2*, **(G)** PAH1OE, and **(H)** PAH2OE. Results for N-sufficient (+N, light gray) and N-depleted (-N, dark gray) conditions are shown. Values represent the mean ± SD of measurements made on samples from three different plants for each genotype.
