# Supplementary figures and images for: Arabidopsis Phosphatidic Acid Phosphohydrolases Are Essential for Growth under Nitrogen-Depleted Conditions
Source: Front Plant Sci. 2017 Oct 31;8:1847. doi: 10.3389/fpls.2017.01847 (PMC5671605; doi:10.3389/fpls.2017.01847)

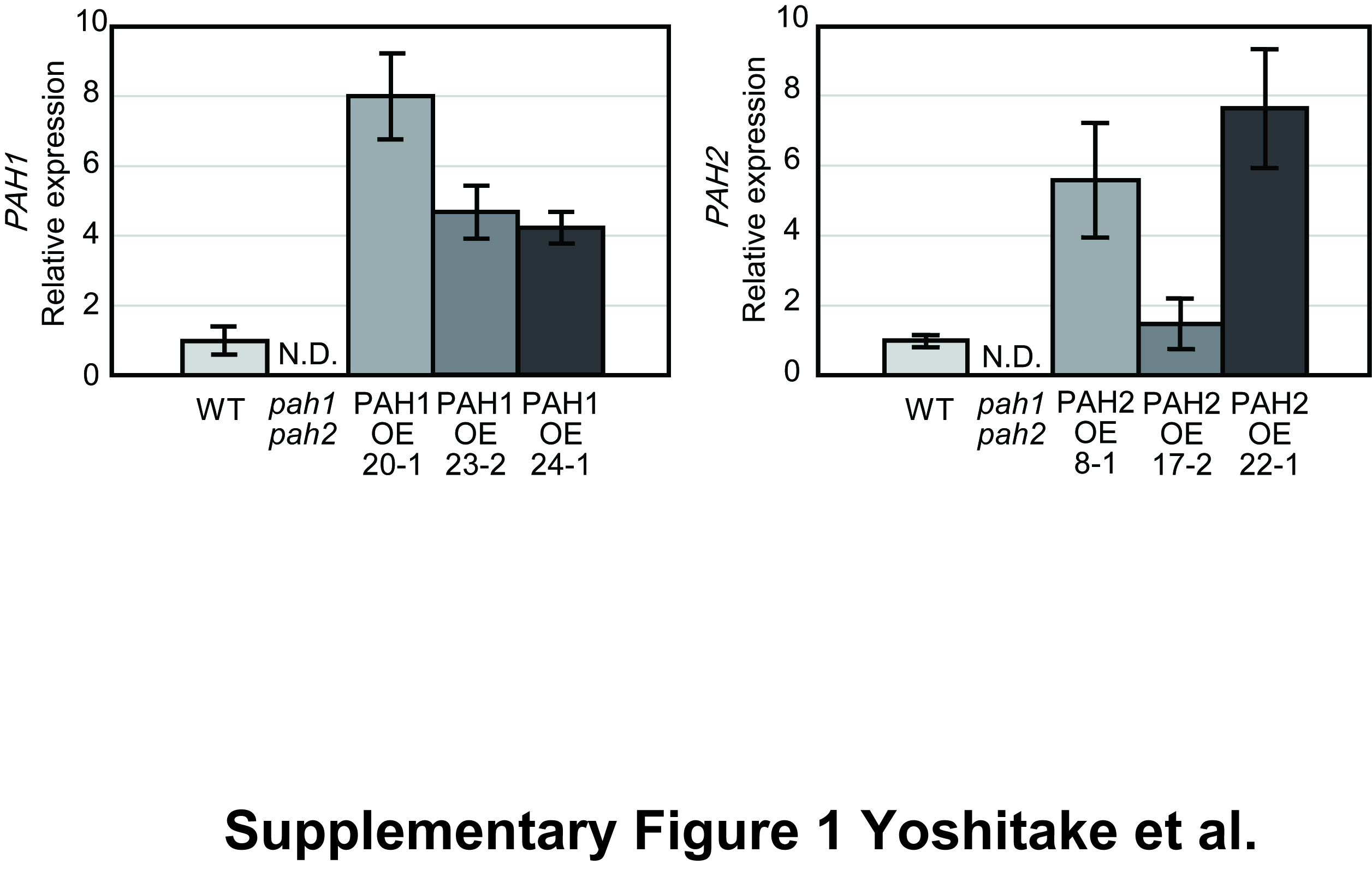

Supplement: FIGURE S1 — Expression levels of PAH1 and PAH2 in WT, pah1 pah2, and the transgenic plants PAH1OE and PAH2OE under N-sufficient conditions. Expression of PAH1 and PAH2 relative to their corresponding levels in WT under N-sufficient conditions as assessed by quantitative reverse transcription–PCR. Values represent the mean ± SD of measurements made on samples from three different plants for each genotype. [file Image_1.jpg]

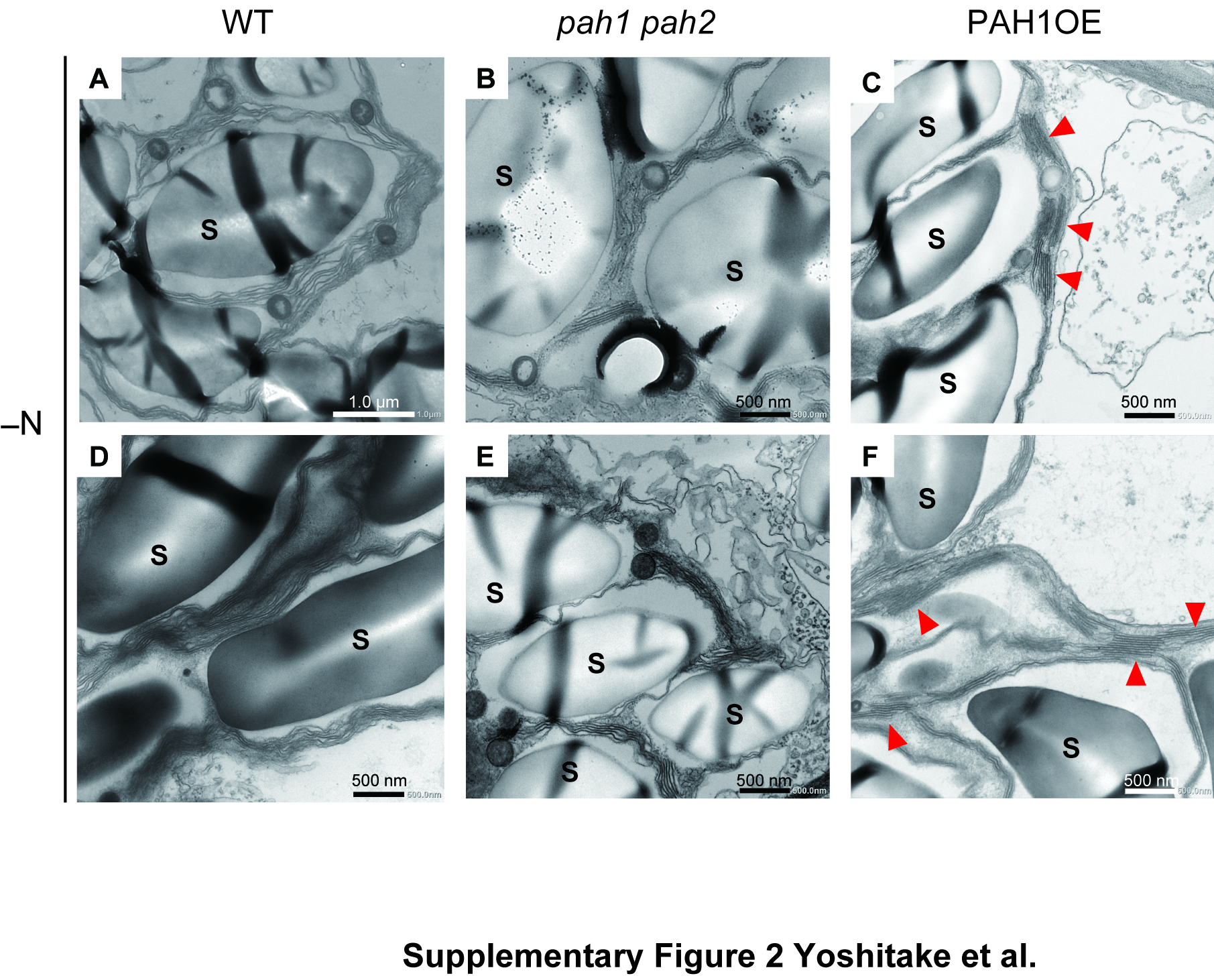

Supplement: FIGURE S2 — Electron microscopy of chloroplast membrane structures in leaves. (A,D) WT, (B,E) pah1 pah2, and (C,F) PAH1OE under N-depleted conditions. Red arrowheads indicate grana-lamellae stacking structures in thylakoid membranes. S, starch granules. [file Image_2.jpg]

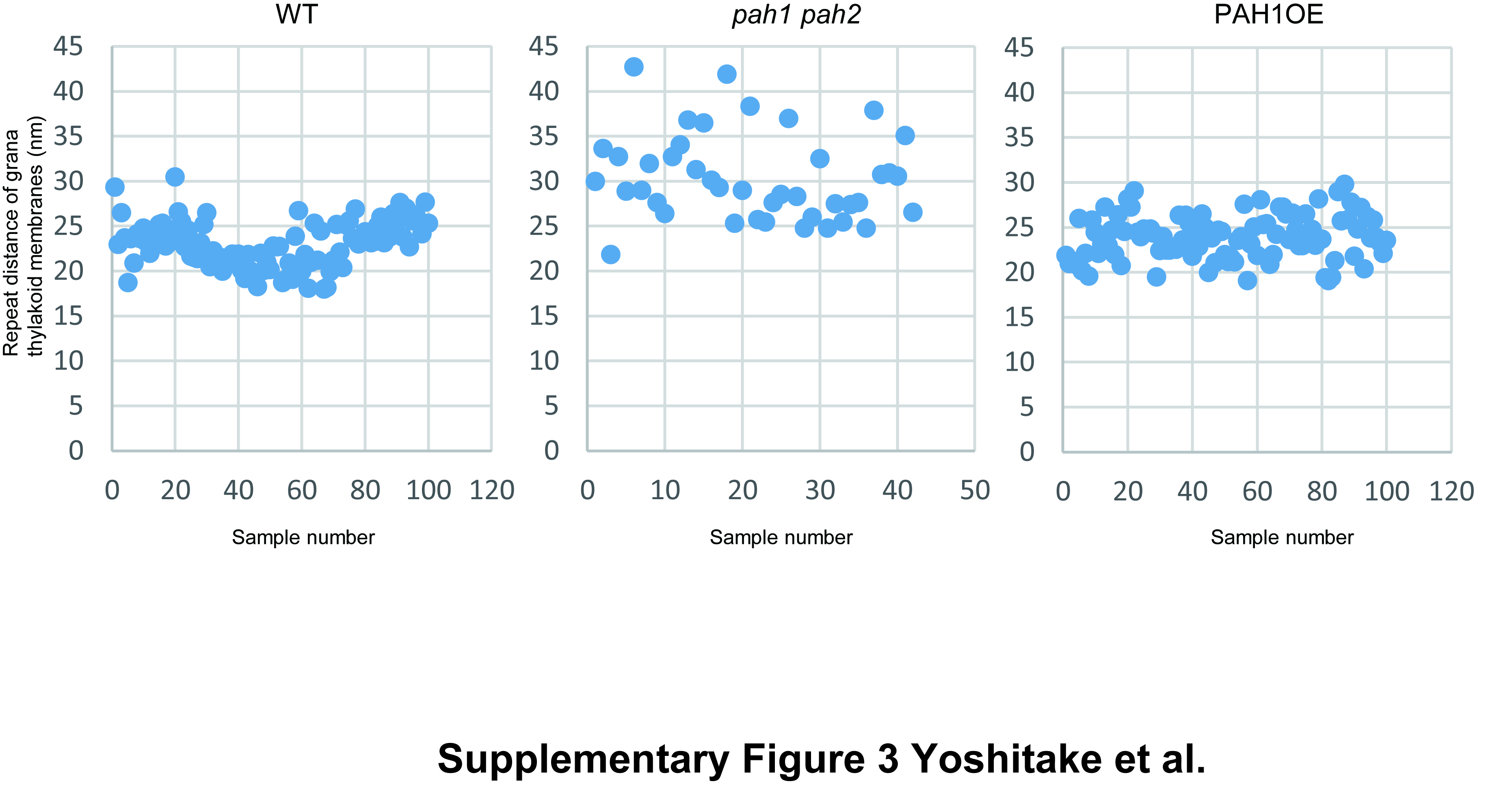

Supplement: FIGURE S3 — Ultrastructural analysis of the stacking repeat distance in thylakoids of WT, pah1 pah2, and PAH1OE under N-depleted conditions. (A) WT, (B) pah1 pah2, (C) PAH1OE. The repeat distance of grana thylakoid membranes was analyzed from transmission electron microscopy images according to the method of Wang et al. (2014). (A) and (C), n = 100 grana thylakoid membrane regions from 10 different chloroplasts, with 10 grana thylakoid membrane regions from each chloroplast; (B), n = 42 grana thylakoid membrane regions from 10 different chloroplasts, with four or five grana thylakoid membrane regions from each chloroplast. [file Image_3.jpg]

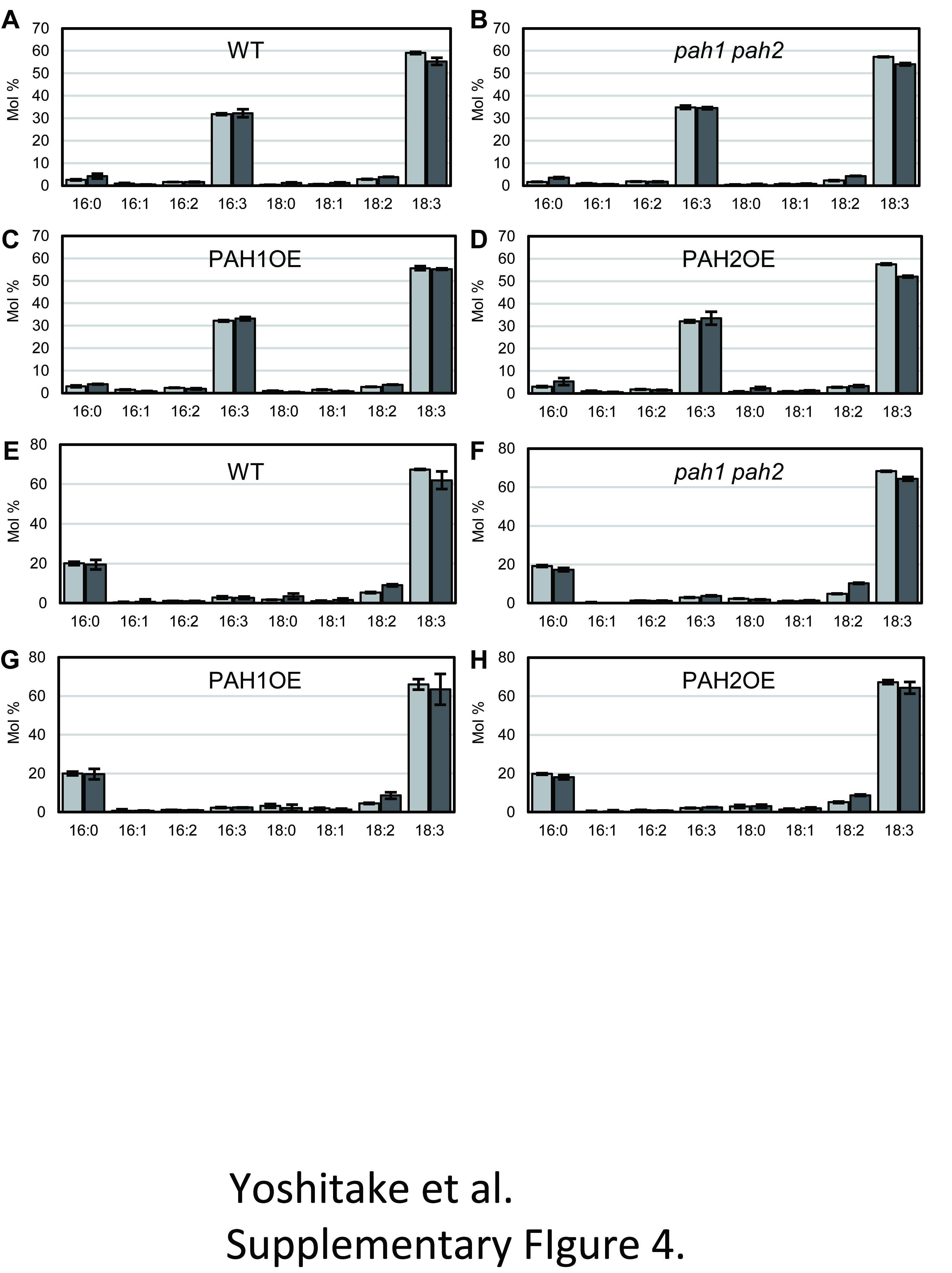

Supplement: FIGURE S4 — Fatty acid compositions of MGDG and DGDG. MGDG in (A) WT, (B) pah1 pah2, (C) PAH1OE, and (D) PAH2OE. DGDG in (E) WT, (F) pah1 pah2, (G) PAH1OE, and (H) PAH2OE. Results for N-sufficient (+N, light gray) and N-depleted (-N, dark gray) conditions are shown. Values represent the mean ± SD of measurements made on samples from three different plants for each genotype. [file Image_4.jpg]

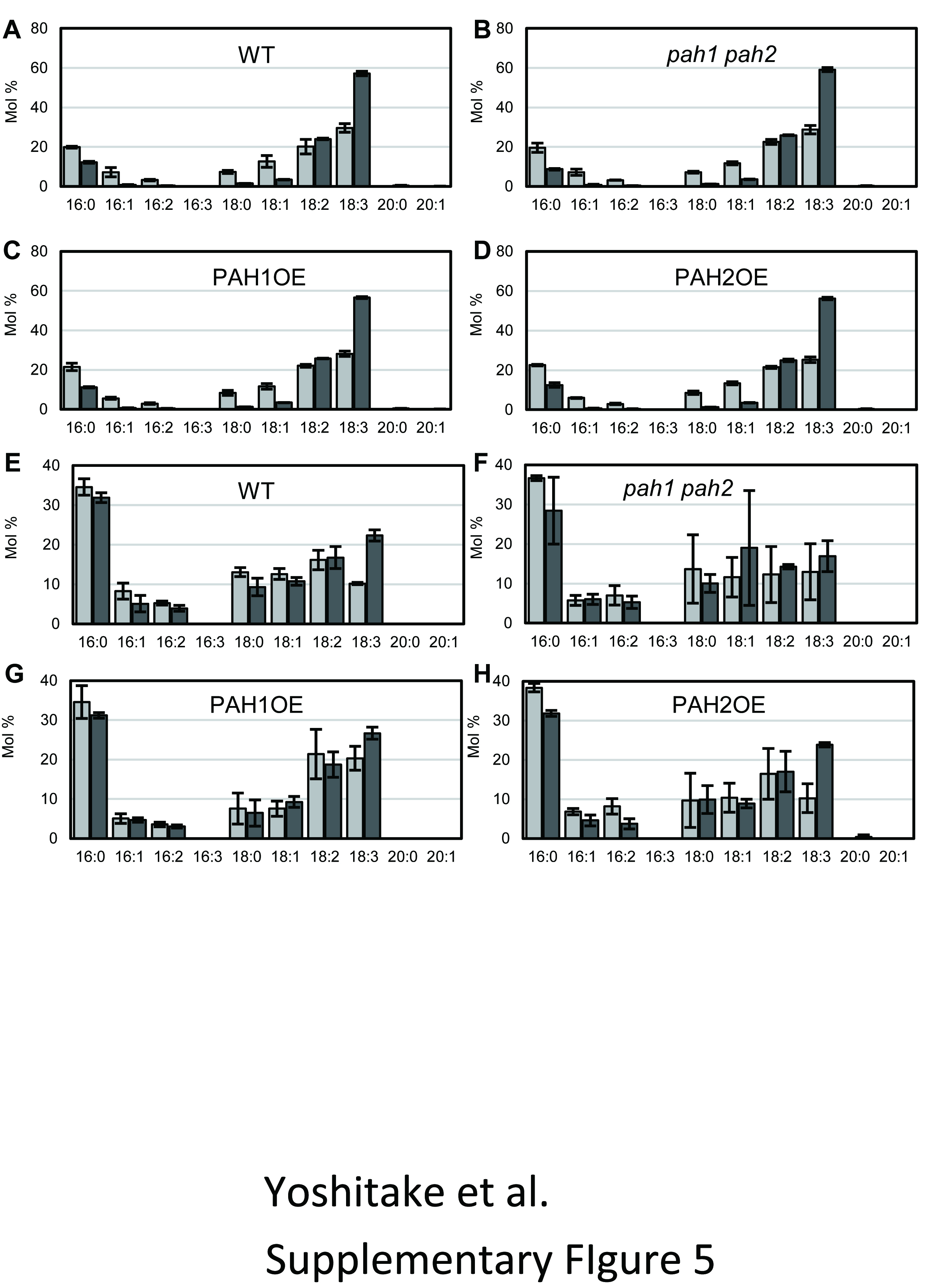

Supplement: FIGURE S5 — Fatty acid compositions of TAG and DAG. TAG in (A) WT, (B) pah1 pah2, (C) PAH1OE, and (D) PAH2OE. DAG in (E) WT, (F) pah1 pah2, (G) PAH1OE, and (H) PAH2OE. Results for N-sufficient (+N, light gray) and N-depleted (-N, dark gray) conditions are shown. Values represent the mean ± SD of measurements made on samples from three different plants for each genotype. [file Image_5.jpg]
